# Design and implementation of multi-band reflectarray metasurface for 5G millimeter wave coverage enhancement

**DOI:** 10.1038/s41598-024-66330-4

**Published:** 2024-07-03

**Authors:** Bilal Tariq Malik, Shahid Khan, Slawomir Koziel

**Affiliations:** 1 Faculty of Electronics, Telecommunications, and Informatics, Gdansk University of Technology, 80-233 Gdańsk, Poland; 2 Engineering Optimization and Modeling Center, Reykjavik University, 101 Reykjavik, Iceland

**Keywords:** Intelligent reflecting surface, Reflectarray metasurface, Millimeter-wave, Coverage enhancement, 5G, Electrical and electronic engineering, Information technology

## Abstract

A compact low-profile multi-band millimeter-wave (mm-wave) reflectarray metasurface design is presented for coverage enhancement in 5G and beyond cellular communication. The proposed single-layer metasurface exhibits a stable reflection response under oblique incidence angles of up to 60$^\circ$ at 24 and 38 GHz, and transmission response at 30 GHz, effectively covering the desired 5G mm-wave frequency bands. The proposed reflectarray metasurface is polarization insensitive and performs equally well under TE and TM polarized incident waves due to the symmetric pattern. In addition, the low profile of the proposed metasurface makes it appropriate for conformal applications. In comparison to the state-of-the-art, the proposed reflectarray metasurface unit cell design is not only compact (3.3 $\times$ 3.3 mm$^2$) but also offers two reflections and one transmission band based on a single-layer structure. It is easy to reconfigure the proposed metasurface unit cell for any other frequency band by adjusting a few design parameters. To validate the concept of coverage enhancement, a 32 $\times$ x32 unit-cell prototype of the proposed reflectarray metasurface is fabricated and measured under different scenarios. The experimental results demonstrate that a promising signal enhancement of 20–25 dB is obtained over the entire 5G mm-wave n258, n259, and n260 frequency bands. The proposed reflectarray metasurface has a high potential for application in mm-wave 5G networks to improve coverage in dead zones or to overcome obstacles that prevent direct communication linkages.

## Introduction

**Figure 1:**
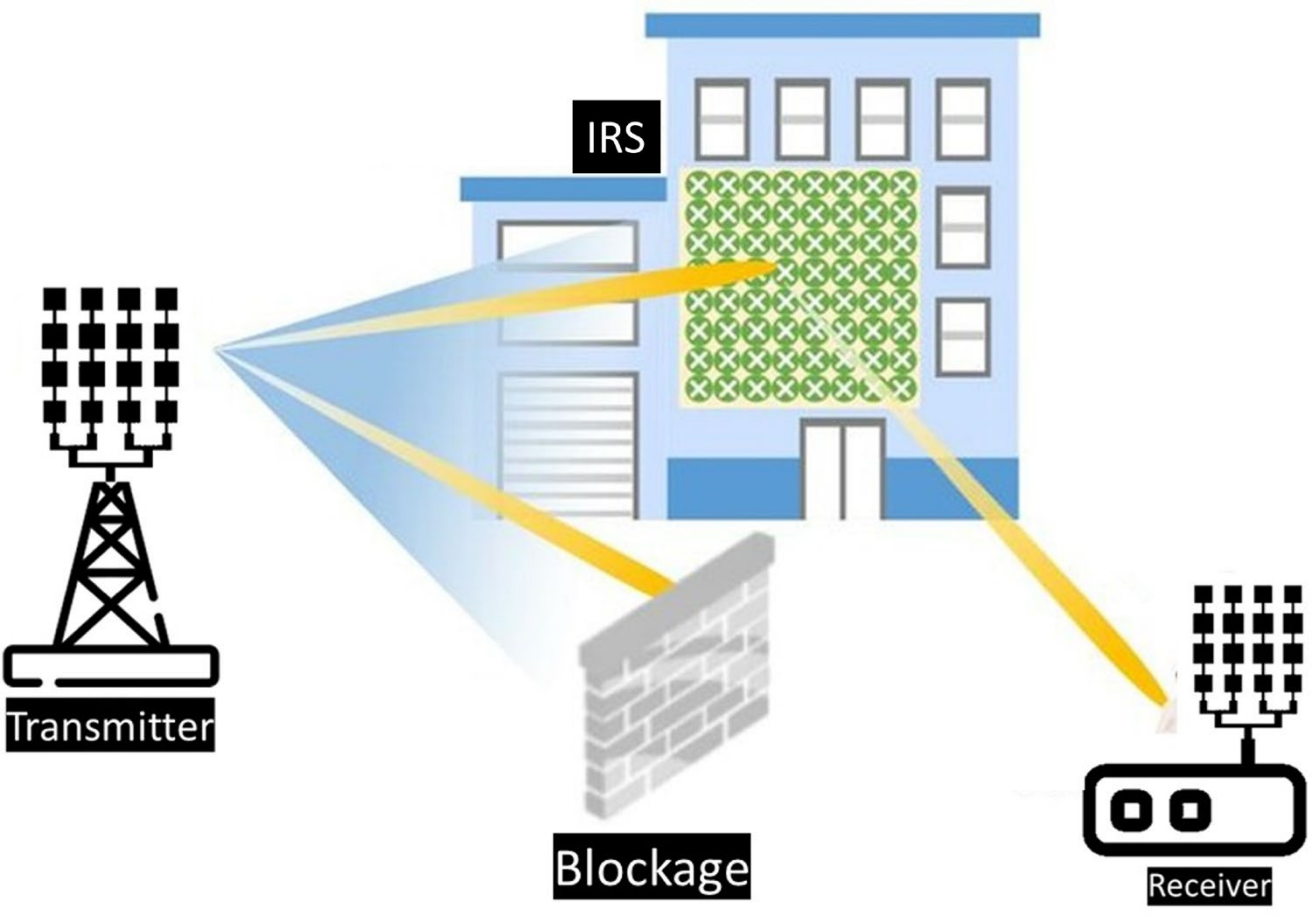
Architecture of IRS implementation for coverage enhancement.

The millimeter-wave frequency bands n257 (26.5–29.5 GHz), n258 (24.25–27.5 GHz), n259 (39.5–43.5 GHz), n260 (37.0–40.0 GHz), and n261 (27.50–28.35 GHz) have been allocated and already been utilized for 5G and beyond communications^1^. The large available bandwidth, high data rate transmission, and low latency are the prominent features of millimeter-wave (mm-wave) frequency bands for 5G and beyond networks. However, mm-waves can be substantially distorted or even blocked by obstacles, resulting in signal blind spots thereby limiting the system to line-of-sight (LOS) communication^2^. When 5G and beyond networks are deployed in indoor environments with a high user density, like conference rooms, stadiums, airports, etc., the walls and enormous obstacles can readily block mm-wave signals, which can lead to coverage degradation and link instability in non-line of sight communications^2^. Intelligent Reflection Surfaces (IRSs), also known as reflectarray metasurfaces, are being suggested as an effective and cheap way to reduce the weakening of signals caused by obstacles which alternatively improve the coverage. They help to create an alternative path for the signal, which maintain a stable communication even with certain blockages, as shown in Fig. 1. Intelligent Reflecting Surfaces has been emerging as a promising technology in 5G mm-wave communications for performance enhancement^1–6^. IRS for mm-wave frequency bands is gaining popularity as a result a significant amount of theoretical work on IRS for coverage enhancement has been reported in the recent literature^4,7–10^. In^4^, a detailed theoretical analysis is presented to show how an IRS is used in wireless communication systems to extend coverage. Multiple deployment scenarios were considered to evaluate the performance improvements through IRS-supported communications. In^7^, the authors theoretically investigated the use of IRS in urban conditions to improve the coverage and capacity of millimeter-wave communication. They demonstrated through extensive simulations and optimizations that the IRS can mitigate the blockage effects and enhance coverage. In^10^, the authors investigated the possibility of expanding the coverage area of millimeter wave Unmanned Aerial Vehicle (UAV) to User Equipment (UE) wireless networks by incorporating IRS. A technique was also suggested to maximize the UAV-to-UE communication channel using IRS. Similarly,^9^ proposed the concept of RIS-assisted UAV communication. However, only a handful of published reports are available showing experimental results and practical implementations of passive IRS, particularly for mm-wave bands, the majority of which have been conducted on the sub-6 GHz band^11–13^. To the best of the author’s knowledge, no published literature has reported the experimental results for coverage enhancement through passive IRS covering all the proposed 5G mm-wave bands (n257, n258, n259, and n260). A practical RIS design and far-field measurements for signal enhancement have been reported in^1^. However, the proposed design is a multilayer complex structure having many vias connecting the patch with the ground, and the design only covers one 5G band at 28 GHz, which cannot fulfill the coverage enhancement requirements for other 5G bands at 38 GHz. In^14^, the design and implementation of passive IRS panels have been presented for performance improvement at 28 GHz 5G applications. In another research work, a mm-wave metasurface was proposed at 28 and 39 GHz for 5G applications. However, it has a two-layer geometry. Each layer contributes to a single band. The major drawback is that the mentioned design does not provide any practical measurements to support the claims on coverage enhancement^15^.

Compared to the current state-of-the-art designs, the proposed unit cell design is notably compact, measuring just 3.3 $\times$ 3.3 mm$^{2}$. This size is smaller than most designs reported in the recent literature that operate within similar frequency bands. Additionally, it achieves multi-band operation while being incorporated into a single-layer structure. This is in contrast to many other designs that require multi-layer structures, which can be complex, bulky, and difficult to implement in conformal applications. Using simple resonant elements in the proposed design allows for easy reconfiguration across different frequency bands by adjusting a few design parameters. The unit cell maintains stability under various angles of incidence up to 60$^\circ$ for both TE and TM polarizations. Its symmetric, low-profile layout ensures polarization insensitivity and excellent performance in conformal conditions. This research is the first of its kind in demonstrating how a simple passive IRS can enhance signal strength across all 5G millimeter-wave frequency bands. Experimental validation shows a significant coverage enhancement by improving millimeter-wave path loss from transmitter to receiver. The experimental results reveal a promising signal enhancement of 20–25 dB across the entire 5G mm-wave n258, n259, and n260 frequency bands. The novelty and the contributions of this research work can be summarized as follows: (i) design and implementation of a compact, low-profile, single-layer passive IRS unit-cell structure for 5G mm-wave applications; (ii) development of a large conformal passive IRS consisting of 32 $\times$ 32 unit-cells for 5G coverage enhancement applications; (iii) experimental demonstration of coverage enhancement at 24 and 38 GHz, not reported before. The structure of this article can be outlined as follows. Section 2 explains the proposed metasurface unit cell design with geometry evolution and parametric analysis of the structure. Section 3 provides the implementation and measurements of the proposed metasurface including the experimental validation of coverage enhancement by using the proposed metasurface. Section 4 concludes this article.

## Proposed reflectarray metasurface design

A metasurface consists of an immense number of passive reflecting elements deployed periodically in a two-dimensional pattern. These elements can effectively manipulate the incident electromagnetic waves and redirect them towards the desired direction. The passive elements of metasurface do not actively amplify the incident wave, unlike antenna arrays. They intelligently manipulate the phase and amplitude of incident signals to enhance or suppress the signal, depending on its design. Metasurfaces can be deployed on walls, buildings, and even outdoor trees for 5G coverage enhancement applications. Therefore, a compact low profile multiband mm-wave reflectarray metasurface with good angular stability and polarization insensitivity is highly desirable for the mentioned applications. In the following sub-sections, an overview of the geometry of the proposed metasurface unit cell is presented. Subsequently, the design evolution along with equivalent circuit and parametric analysis of the proposed unit cell, followed by a detailed performance evaluation based on the surface current distribution, angle of incidence, polarization, and conformability of structure is also presented.

**Figure 2:**
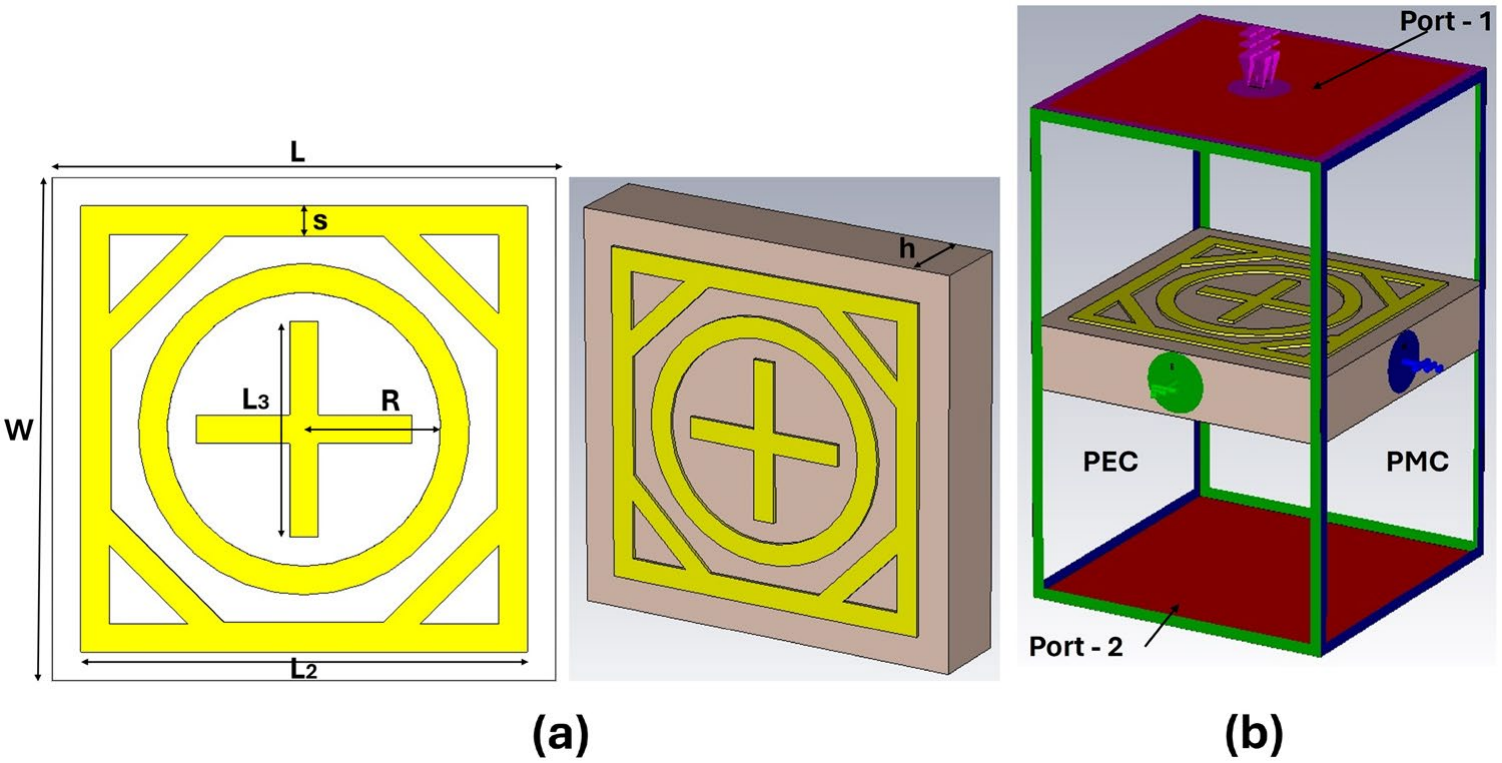
Proposed unit cell: (**a**) parameterized geometry, (**b**) simulation model with boundary conditions.

### Unit cell design

This section elaborates on the design process of the proposed metasurface unit cell. In the literature, various elements such as N-poles, loop-shaped or patch-shaped components, and combinations of these have been used to design metasurfaces depending on the intended application^16^, In this research article, we choose a combination of square and circular loop structures for the proposed metasurface due to their structural simplicity, good angular and polarization stability, and wideband response^16^. In this arrangement, multiple loops for multi-band operations can be efficiently organized while ensuring a small footprint, allowing the arrangement of large numbers of unit cells in a compact space^17^. The geometrical configuration of the proposed single-layer dual-band metasurface unit cell is presented in Figure 2(a). The dielectric substrate used in this work is Arlon AD 250, with a thickness of 0.76 mm, a dielectric constant of 2.5, and loss tangent tan$\delta$ = 0.0013. The proposed unit cell consists of a single copper layer, which is on the top side of the dielectric substrate, while the lower side of the dielectric substrate does not contain any copper layer. The design parameters of the proposed unit cell are the length of outer square loop $L_2$, the radius of inner circular *R*, and the dimensions of cross dipole $L_3$ as depicted in Fig. 2a. CST Microwave Studio is utilized to design and simulate the proposed IRS. The unit cell periodic boundary conditions are assigned along the *x* and *y*-axis, and the incident wave is applied along the *z*-axis, as shown in Fig. 2b.

**Figure 3:**
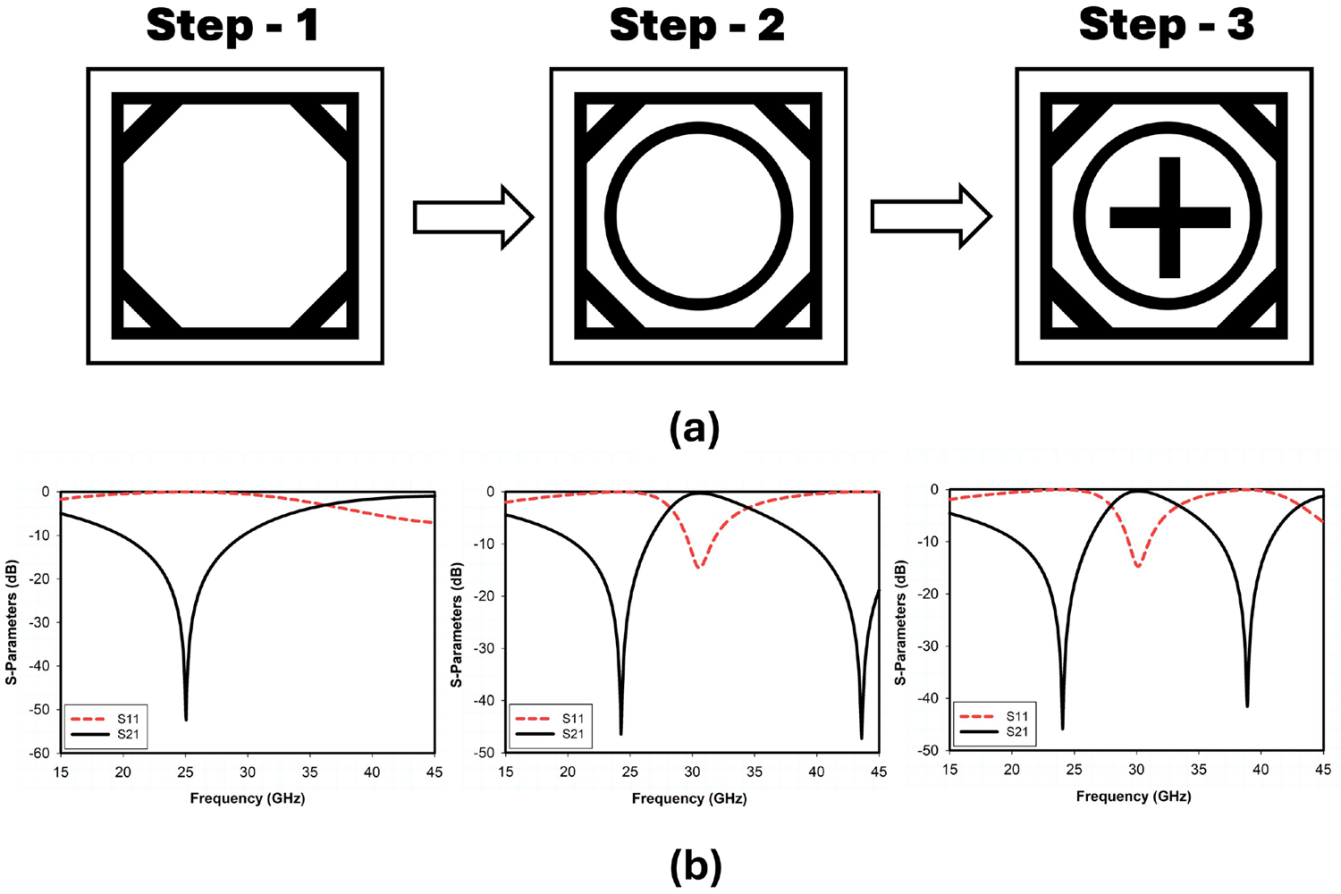
Evolution of the proposed unit cell: (**a**) cell geometries at subsequent design steps, (**b**) corresponding reflection and transmission coefficients.

**Figure 4:**
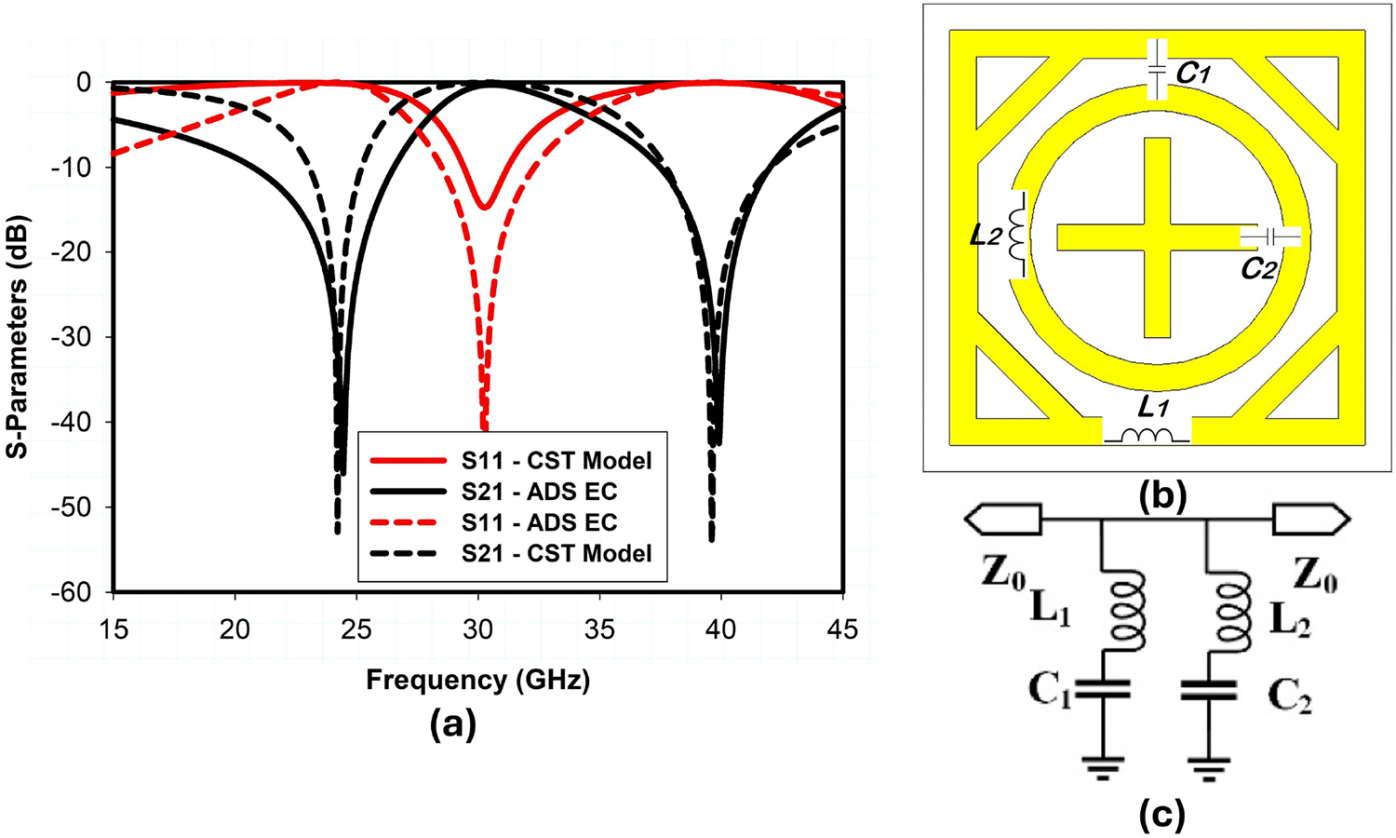
Equivalent circuit of the proposed unit cell: (**a**) S-Parameters, (**b**) unit cell model in CST, (**c**) equivalent circuit in ADS ($L_1$ = 0.61nH, $C_1$ = 0.07pF, $L_2$ = 0.31nH, $C_2$ = 0.05pF).

**Figure 5:**
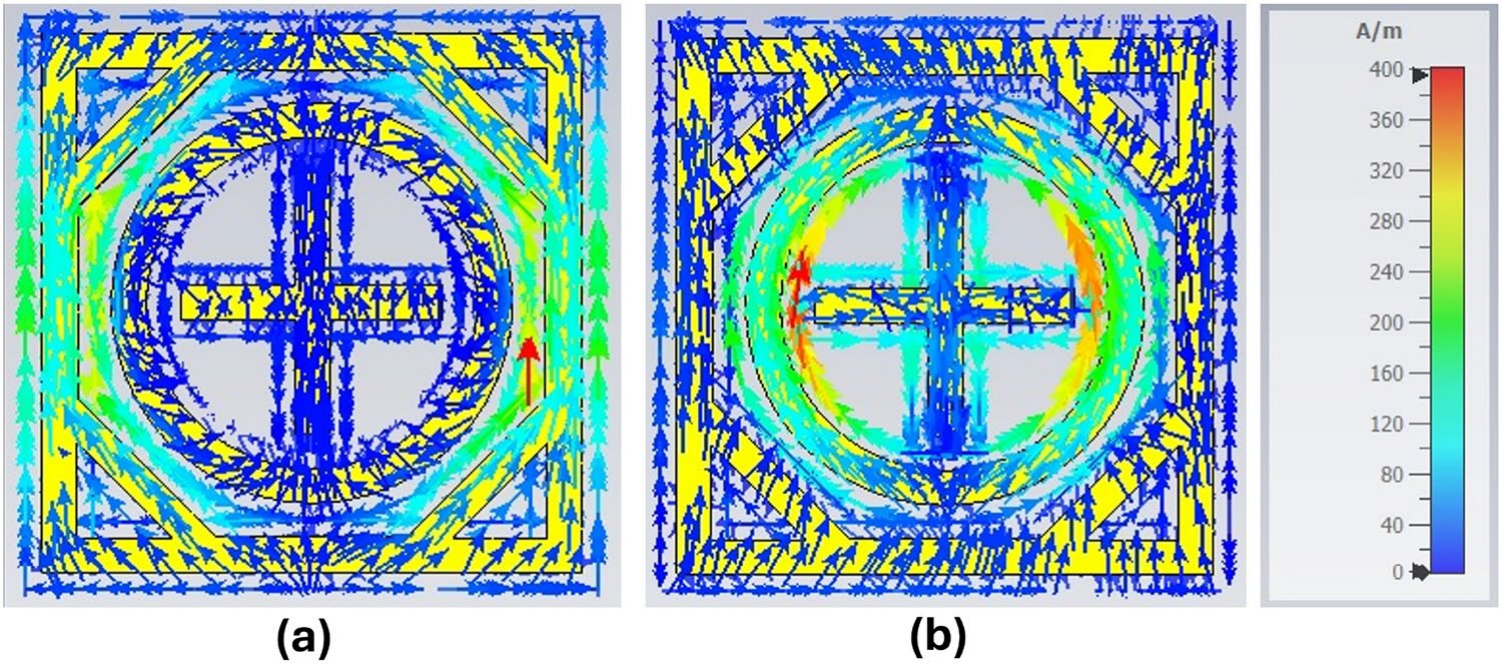
Surface current distribution, (**a**) at 24 GHz, (**b**) at 38 GHz.

### Unit cell design evolution

The stepwise geometry evolution of the proposed unit cell is presented in Fig. 3a. The corresponding reflection and transmission coefficient plots for the stepwise design of the unit cell are shown in Fig. 3b. The proposed metasurface design incorporates multiple meta-surface structures (square loop, octagonal loop, circular loop, and cross dipole) for enhancement of design flexibility and scalability in terms of multiple frequency bands. The outer square loop ($L_2$) is responsible for the lower-frequency band as shown in the corresponding S-parameters response of Step-1, and the upper-frequency band mainly depends on the inner circular ring (*R*) as shown in the s-parameters response of Step-2. The structure obtained in Step-2 is not properly tuned at the desired 5G mm-wave n259 and n260 band and is resonating at a frequency of 44 GHz. When attempting to relocate the upper-frequency band from 44 GHz to 38 GHz by adjusting/increasing the dimensions of the circular ring, we will also need to increase the dimensions of the outer square ring. Consequently, the lower band, currently tuned to the desired 5G frequency of 24 GHz, will shift to a lower frequency, moving away from the desired range. To maintain the lower band at 24 GHz, the outer square ring’s ($L_2$) dimensions must remain fixed at 3.1mm. This constraint prevents further enlargement of the circular ring (*R*) to adjust the upper band. Therefore, to achieve resonance at 38 GHz for the upper-frequency band, it becomes essential to introduce a cross dipole in step-3. The coupling between the circular ring and the cross dipole allows precise tuning without altering the fixed dimensions of the outer square ring, ensuring both frequency bands are accurately targeted for 5G applications. The corresponding transmission and reflection curve response of Step-3 depicts that the structure resonates at the desired 5G band. It is also incorporated with an octagonal loop meta-surface, which enhances the bandwidth by reducing the coupling between surrounding metasurface structures. The proposed integrated loop structure considerably reduces signal degradation and improves its overall performance.

The proposed single-layer metasurface structure offers two reflection bands (24 and 38 GHz) and a single transmission band (30 GHz), which are suitable for millimeter-wave 5G and beyond applications. Figure 3 shows that the proposed reflecting metasurface incorporates two reflection bands ranging from 19.5 to 26.2 GHz and 37.1 to 41.8 GHz with fractional bandwidths of 27 and 12.5, respectively, and one transmission band from 29.5 to 31.2 GHz. It would be an ideal candidate for all the desired mm-wave 5G bands.

The equivalent circuit is authenticated using the advanced design system (ADS) software and compared to CST-simulated results. $L_1$ and $L_2$ are the self-inductance of the outer square loop and the inner circular ring, $C_1$ is the capacitance between the adjacent square and circular ring element, and $C_2$ is the capacitance between the adjacent inner circular ring and cross dipole element as shown in Fig. 4b. Therefore, through proper adjustment of $C_1$, $L_1$, $C_2$, and $L_2$ one can relocate the lower ($f_1$) and upper ($f_2$) resonant frequencies of the proposed metasurface as follows

1 $$\begin{aligned} f_n=1/\left( 2\pi \sqrt{(L_n C_n )}\right) ,n=1,2 \end{aligned}$$

The simplified equivalent circuit of the proposed unit cell is shown in Fig. 4c. As discussed earlier, the outer square ring corresponds to the lower frequency band, and the inner circular ring corresponds to the upper resonant frequency. Therefore, the lumped components $L_1$, $C_1$, and $L_2$, $C_2$ correspond to the lower and upper resonant frequency, respectively, as shown in Fig. 4b. The simulated |*S*11| and |*S*21| of the proposed metasurface unit cell model in CST and the equivalent circuit model (ECM) in ADS are presented in Fig. 4a, which are well aligned. The ADS results appear better than the CST model in terms of impedance matching because the ECM consists of lossless lumped components, unlike the CST model. The surface current distribution of the TE-polarized incident EM wave is presented in Fig. 5. In Fig. 5a, the surface current has a higher density at the outer square ring of the unit cell, therefore, the outer square ring mainly constitutes the lower reflection band at 24 GHz. On the other hand, a higher current density appears around the inner circular ring as well as on the cross dipole, which shows the upper resonant frequency at 38 GHz is primarily controlled by the coupling between the circular ring and cross dipole as illustrated in Fig. 5b.

**Figure 6:**
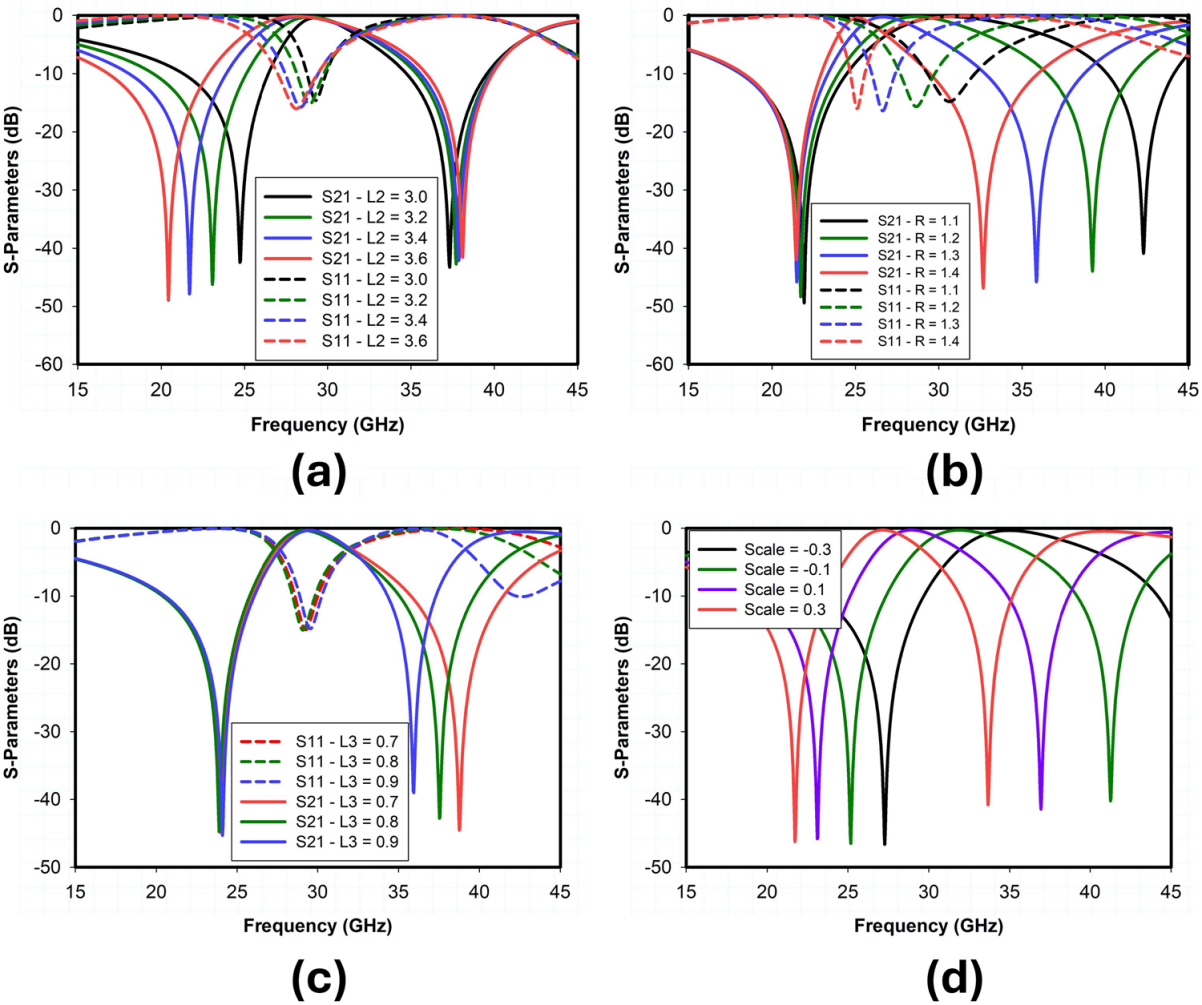
Parametric analysis on the proposed unit cell.

**Figure 7:**
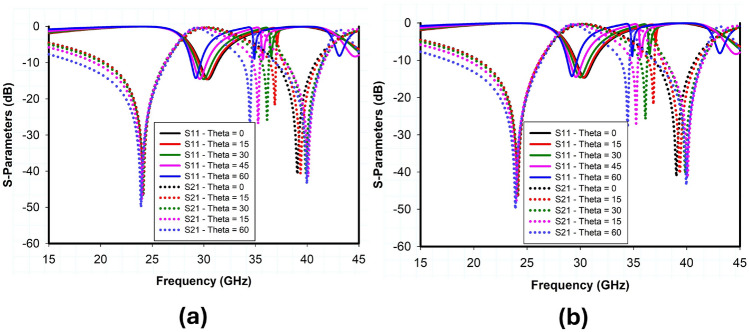
S-parameter response with different angle of incidence a) T.E. Polarization b) T.M. Polarization.

**Figure 8:**
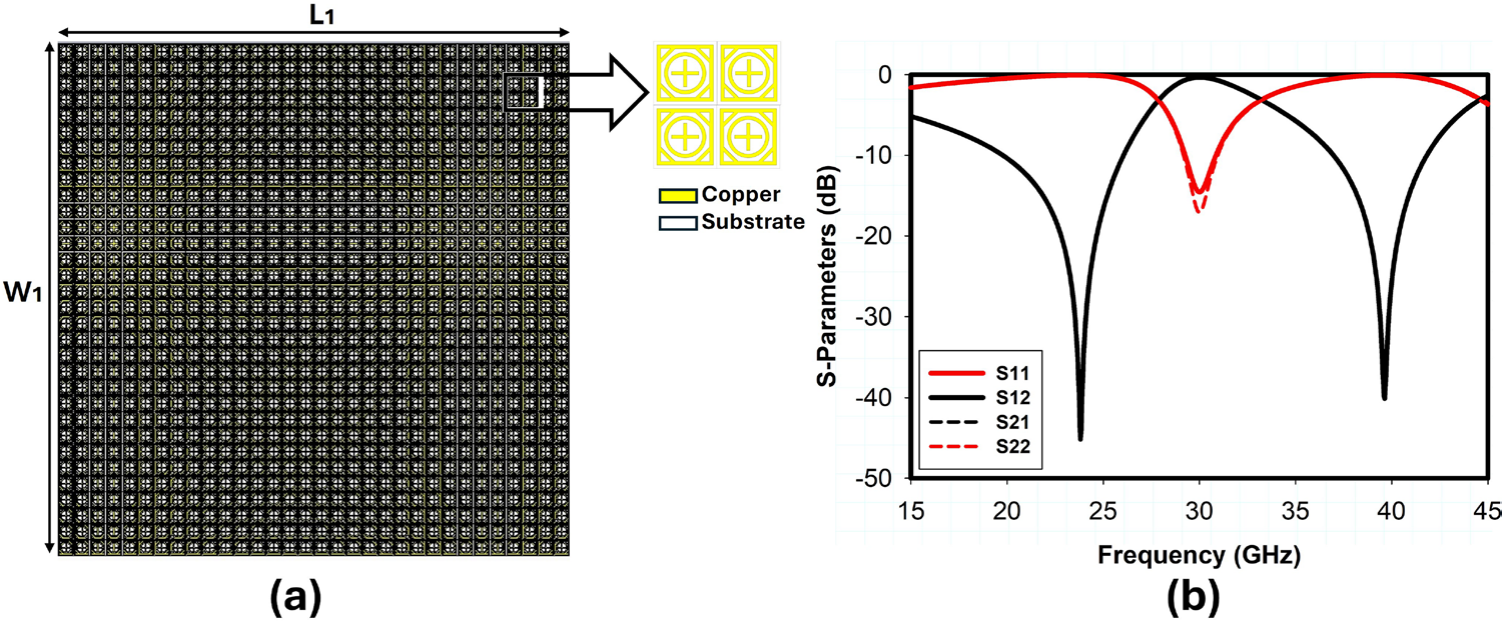
32x32 Unit cell passive IRS design: (**a**) simulation model, (**b**) S-parameters.

### Parametric analysis

In this section, a detailed parametric analysis of the proposed metasurface unit cell design is conducted. Extensive full-wave simulations have been carried out to analyze and optimize each parameter of the proposed unit cell. These parameters include the dimensions of the outer square loop ($L_2$), the radius of the inner circular loop (*R*), and the length of the cross dipole ($L_3$). The impact of these parameters on the reflection and transmission response of the proposed unit cell has been studied. In unit cell design, the outer square loop and inner circular loop is used to control the position of the lower and upper reflection bands respectively. The cross dipole controls the gap between the upper and lower reflection bands while maintain the overall size of the structure. To demonstrate the previous statement, Fig. 6 depicts the results of a parametric analysis of the geometric variables $L_2$, $L_3$, and *R*.

**Figure 9:**
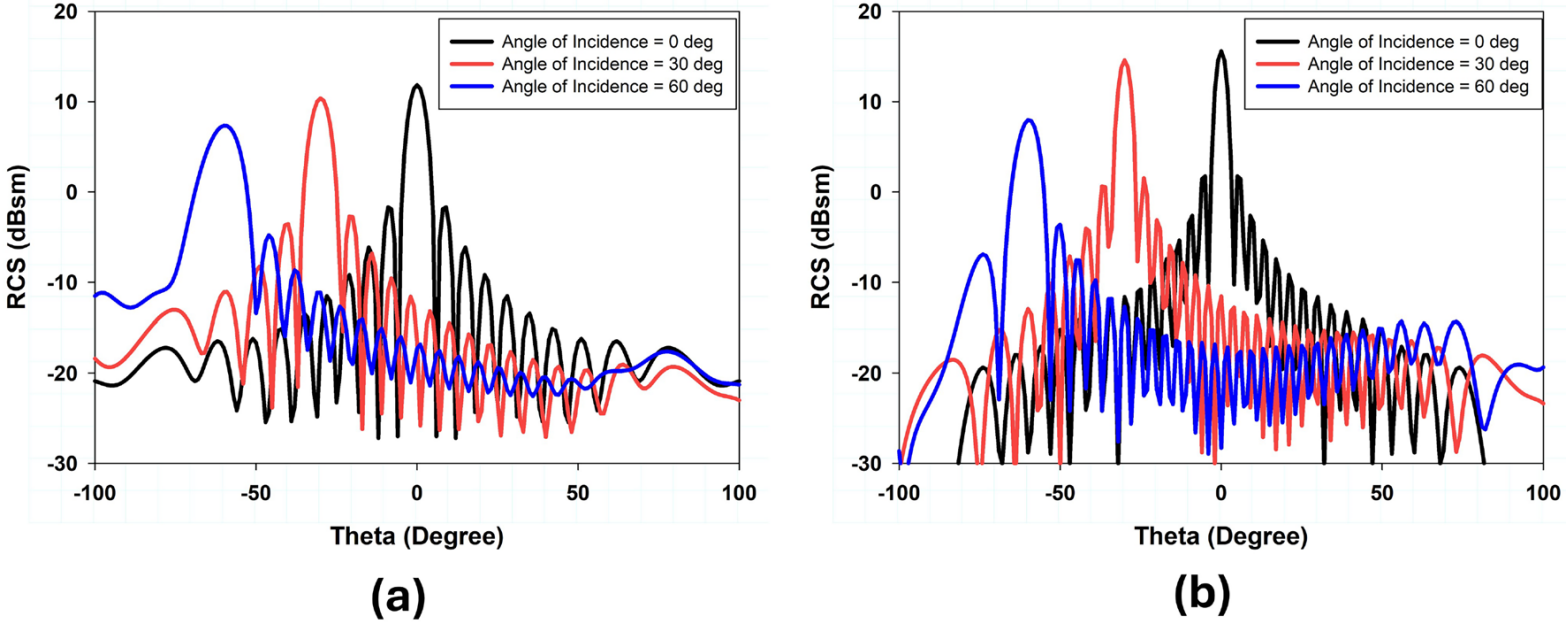
Simulated RCS response of angle reciprocity for different angles of incidence (**a**) 24 GHz (**b**) 38 GHz.

**Figure 10:**
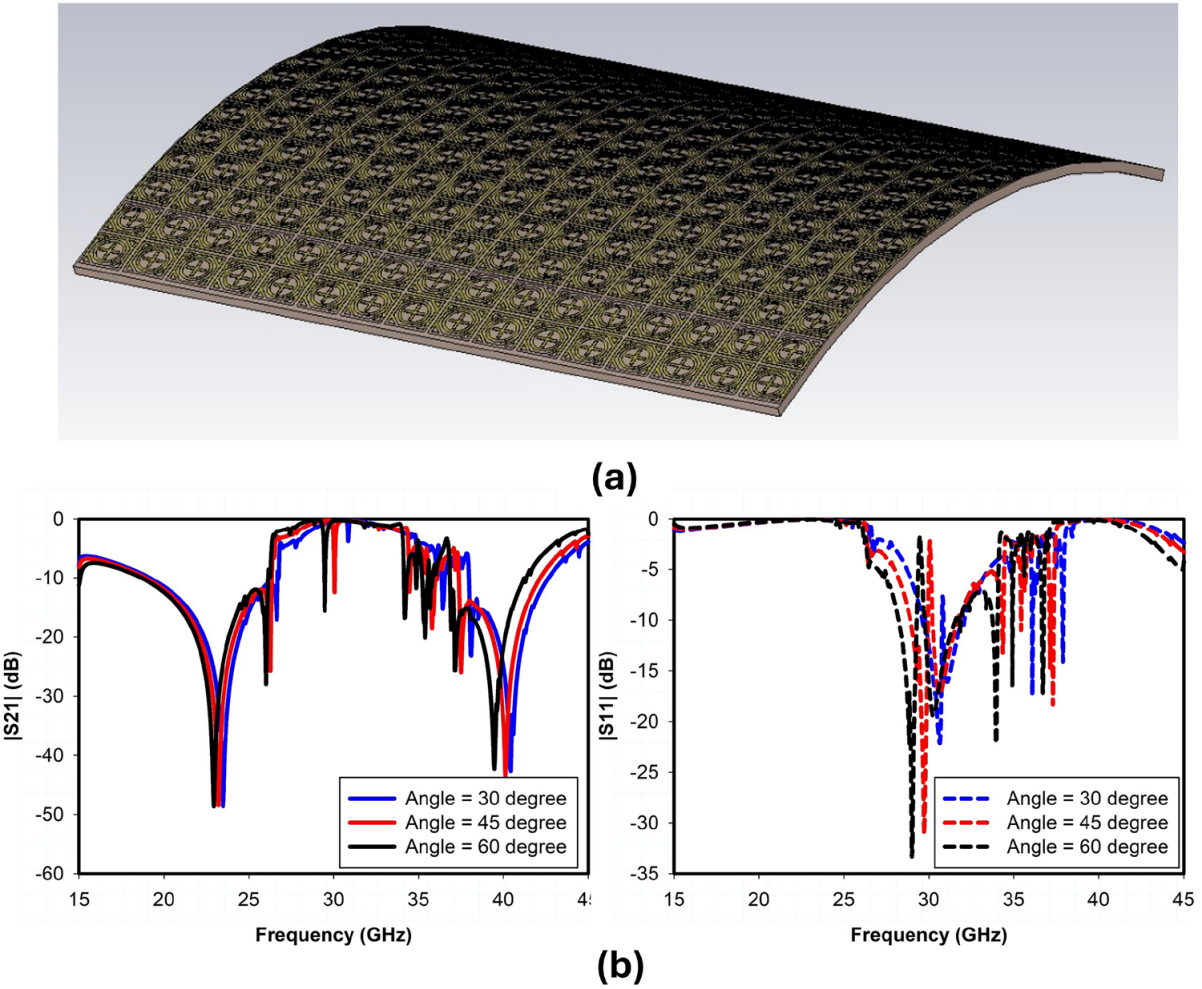
Conformal Model of passive IRS, (**a**) Simulation Model, (**b**) S-Parameters.

As shown in Fig. 6a, changing the value of variable $L_2$ from 3 to 3.6 mm, the resonant frequency of the lower reflection band shifts from 25 to 20 GHz accordingly. On the other hand, if the value of $L_2$ is fixed at 3.4 mm and the dimension of *R* varies from 1.1 to 1.4 mm, the upper reflection band shifts from 42 to 33 GHz, as shown in Fig. 6b. Note that the variation of $L_2$ has almost no effect on the position of the upper reflection band at 38 GHz and transmission band at 30 GHz. Similarly, the dimensions of parameter *R* have no impact on the lower reflection band at 24 GHz, but the transmission band shifts from 30 to 24 GHz if the *R* varies from 1.1 to 1.4 mm. Fig. 6c shows the impact of cross-dipole $L_3$ on the transmission and reflection response of the proposed unit cell. The dimensions of $L_3$ control the position of the upper reflection band without affecting the lower reflection band and transmission band. The upper reflection band and transmission band can be tuned to desired frequency bands by simultaneously optimizing the values of *R* and $L_3$. The parametric analysis indicates that the reflection and transmission bands of the proposed metasurface unit cell are independently as well as entirely scalable by using a scaling factor, as shown in Fig. 6d. The optimized design parameters of the proposed unit cell are summarized in Table 1.

**Table 1:** Optimized design parameters of proposed IRS.

Parameters	Values (mm)	Parameters	Values (mm)
*L*	3.3	*W*	3.3
$L_2$	3.1	$L_3$	1.5
*R*	1.15	*s*	0.2
*h*	0.76	*d*	0.2
$L_1$	118	$W_1$	118

The incident angle stability and polarization insensitivity performance of the proposed design are presented in Fig. 7 through simulated transmission and reflection coefficients obtained using CST Microwave Studio. Based on the data, it is concluded that when the incident angle of electromagnetic waves varies within a range of $\pm 60^\circ$, the transmission and reflection coefficient for both TE and TM polarizations is comparatively stable because of the structure’s symmetry. A 32x32 array of unit cells was also designed and simulated to further validate the performance of large reflectarray metasurface design, as shown in Fig. 8. The RCS response of the proposed metasurface shows the angle reciprocity for different incidence angles at 24 and 38 GHz as depicted in Fig. 9. It shows that the proposed metasurface holds a wide and stable angle-reciprocity up to 60$^\circ$ of the angle of incidence. The proposed structure is also ideal for conformal applications due to its thin and flexible substrate. The conformity of the proposed metasurface is studied under various bending situations, and its transmission and reflection response is stable as shown in Fig. 10.

**Figure 11:**
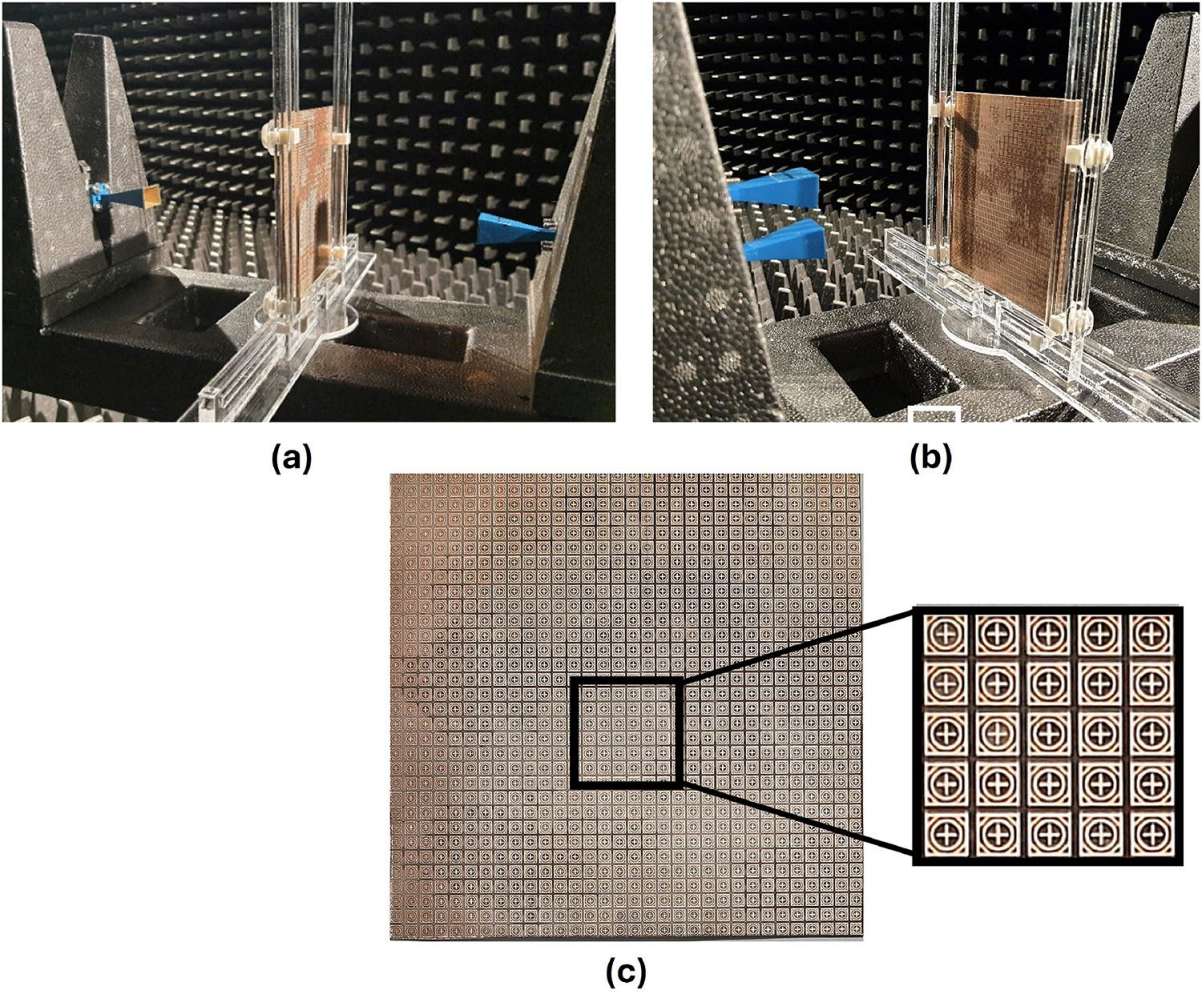
Measurement setup: (**a**) transmission, (**b**) reflection, (**c**) fabricated prototype.

**Figure 12:**
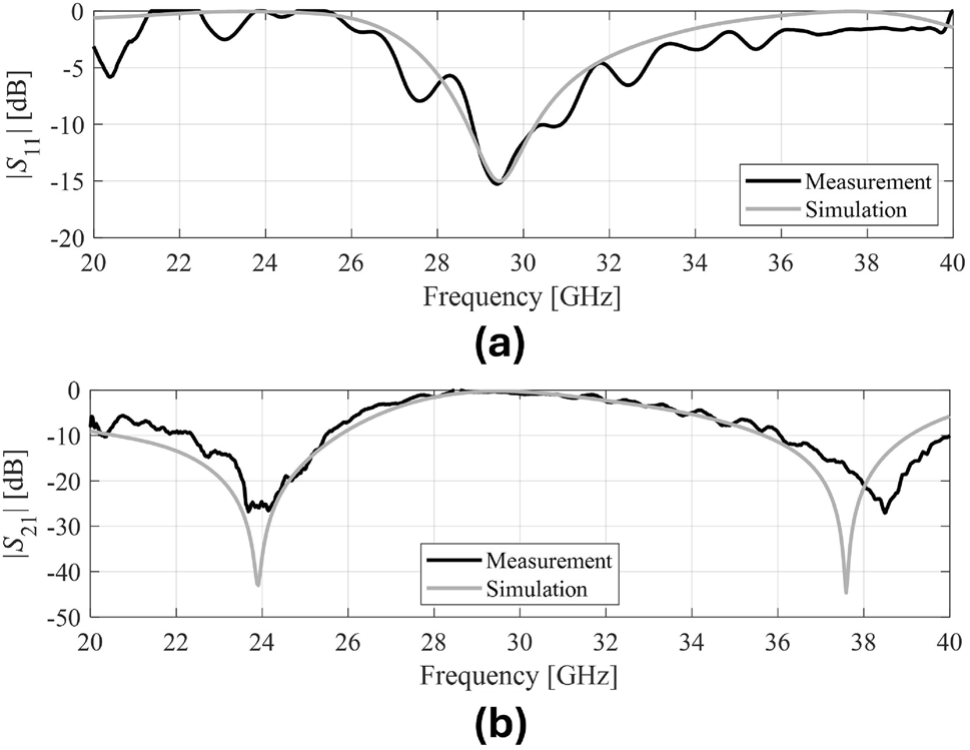
S-parameter measurements: (**a**) reflection, (**b**) transmission.

## Experimental validation and benchmarking

This section presents the measurement results obtained for the fabricated prototype along with the experimental demonstration of coverage enhancement for 5G mm-wave bands. Following an extensive simulation, a prototype of the proposed passive IRS has been fabricated and measured. The proposed passive IRS is made up of a 32 $\times$ 32 unit-cell array and has a total size of 118 $\times$ 118 mm$^2$. The reflection (S11) and transmission (S21) coefficient of the proposed design is measured in an anechoic chamber and the measurement setup is shown in Fig. 11a, b respectively. The top view of the fabricated passive IRS prototype is depicted in Fig. 11c. Two standard gain horn antennas are used for transmission and reception of incident waves, and the proposed passive IRS is placed between the horn antennas, as shown in Fig. 11a, b. The comparison of simulated and measured transmission and reflection coefficients of the proposed passive IRS are presented in Fig. 12a, b, respectively. The measured and simulated S-parameter responses are well-consistent. However, the upper reflection band at 38 GHz slightly shifts toward the right due to fabrication and measurement inaccuracies.

**Figure 13:**
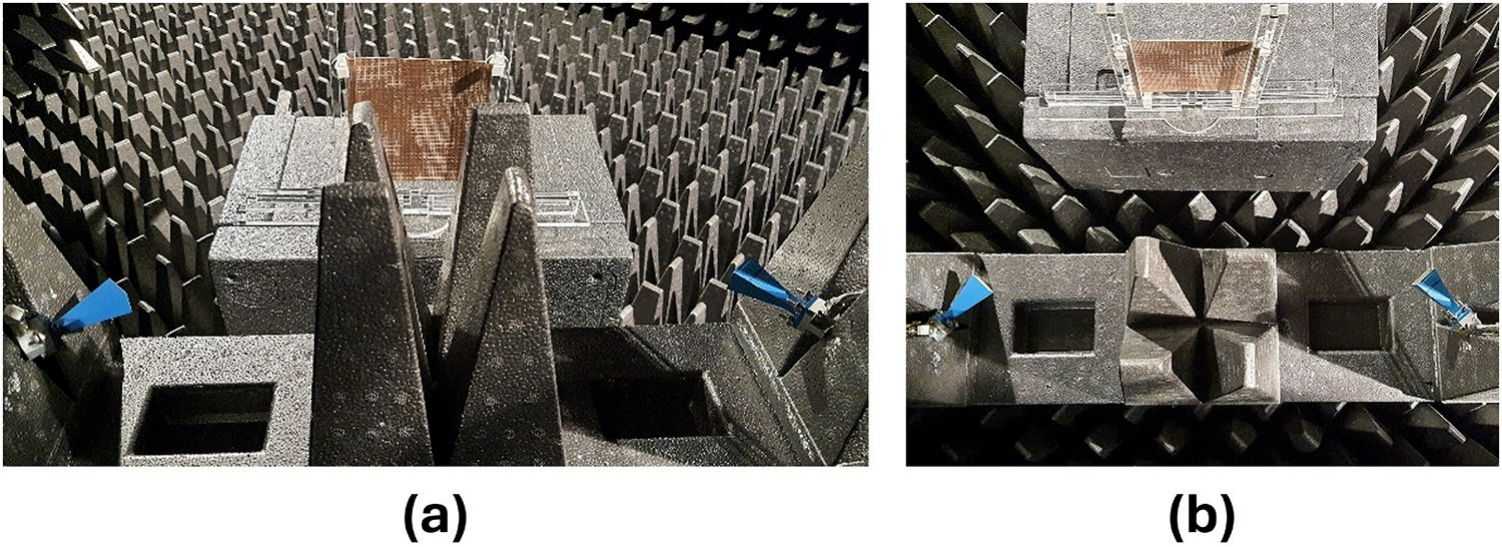
Coverage enhancement measurements, (**a**) Side View, (**b**) Top View.

**Figure 14:**
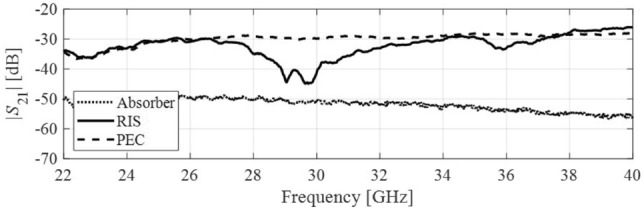
Transmission measurements with and with Proposed IRS.

### Coverage enhancement measurements

This section includes the experimental results demonstrating how the proposed passive IRS can be used to improve the coverage for mm-wave 5G communications. Figure 13 depicts the measurement setup to experimentally validate the concept of coverage enhancement at desired 5G mm-wave frequency bands. In this measurement setup, two standard gain horn antennas are used to transmit antenna Tx and receive antenna Rx. There is an absorber/obstacle placed between the Tx and Rx to block the direct line-of-sight (LOS) path. The proposed passive IRS prototype is placed in parallel to Tx and Rx to avoid the blockage and to provide a second path for transmission, as shown in Fig. 13. The measured transmission coefficient (S21) between the Tx and Rx is presented in Fig. 14. This experimental result represents the comparison of received signal strength at the Rx antenna with an absorber between the Tx and Rx, the proposed passive IRS, and a metallic sheet that has the same size as the IRS aperture. A significant improvement from −52 to −32 dB (20 dB enhancement) is observed at the lower reflection band (24-27 GHz) and from −55 to −25 (30 dB enhancement) at the upper reflection band (37-40 GHz) when the PEC is replaced with proposed passive IRS. It is also notable that the proposed IRS has a transmission band at 30 GHz, on which there is no signal improvement. Based on the experimental findings, it can be concluded that the proposed design provides a promising signal enhancement of 20–25 dB over the entire 5G mm-wave n258, n259, and n260 frequency bands. These experimental results also indicate that the proposed passive IRS design has the potential to improve the 5G connectivity between internet-of-things (IoT) devices.

Table 2 presents a detailed comparison of the proposed passive IRS with the existing designs reported in the recent literature. In^15^, a dual-band FSS was designed at mm-wave 28 and 38 GHz frequency bands for 5G shielding applications. It is a two-layer structure with a unit cell size of 4 $\times$ 4 mm$^2$. In another research work^18^, a reflectarray at 28/38 GHz was proposed for a 5G application without supporting any fabrication prototype and measured results. The discussed design is non-conformal and achieved an incident angle stability of up to $45^\circ$. Similarly, a broadband conformal FSS for 5G shielding application was proposed in^6^. It has a single stopband that covers from 23.92 to 45.9 GHz. Moreover, the metasurfaces presented in^1,3,14^ cover only a single 5G band. Another published article demonstrates a wide reflection band covering 5G mmW frequency bands^19^. However, it is a multilayer structure and is also not suitable for conformal applications. In contrast, the proposed unit cell geometry is compact compared to the benchmark structures^6,15,20–22^. The incident angle stability of the proposed passive IRS is up to $60^\circ$ with polarization-insensitive, which is better than the works reported in^1,2,23^. The critical contributions of the proposed IRS include single-layer miniatured unit cells with a symmetric rotational geometry which makes it easy to design and rescale. The low-profile structure of the proposed IRS is also suitable for conformal multiband applications. The two reflection bands (24 and 38 GHz), and one transmission band (30 GHz) are experimentally verified, showing high stability for the coverage enhancement through passive IRS at 5G n258, n259, and n260 bands.

**Table 2:** Comparison of proposed research with related work in open literature. .

Ref.	Unit-Cell Type	Layer	Substrate/thickness (mm)	Unit-cell size (mm)	Conformal	5G Bands (GHz)	Polarization insensitivity	Signal enhancement (dB)
^15^	Reflect-array	Two	Rogers5880/ 0.254	4 × 4	Yes	28, 38	Yes	N/A
^18^	Reflect-array	Single	Rogers3035/ 0.75	4 × 4	No	28, 39	Yes	N/A
^6^	Reflect-array	Two	Rogers5880/ 0.79	3 × 3	Yes	28	Yes	N/A
^21^	Reflect-array	Two	Rogers4003/ 0.51	4.2 × 4.2	No	28	No	N/A
^20^	Reflect-array	Single	Rogers5880/ 0.79	5 × 5	No	26	No	N/A
^1^	Reflect-array	Multi	Rogers5880/ RF4/ 1.8	3.9 × 3.9	No	28	No	15–20
^24^	Reflect-array	Two	Rogers5880/ 0.79	2.7 × 2.7	No	24	No	N/A
^22^	Reflect-array	Multi	SCGA-500/ 0.76	4.6 × 4.6	No	26, 38	No	N/A
Proposed	Reflect/ Transmit-array	Single	Arlon-AD 250/ 0.76	3.3 × 3.3	Yes	24, 38	Yes	20–25

## Conclusion

This paper presented a novel compact multi-band IRS design for coverage enhancement at mm-wave 5G n258, n259, and n260 bands. According to the authors’ knowledge, no published work has reported the experimental demonstration of coverage enhancement for all 5G mm-wave bands at 24 and 38 GHz. The proposed simple single-layered IRS unit-cell design is not only compact compared to state-of-the-art but also polarization insensitive and has performed equally under oblique incidence angles of up to 60$^\circ$ at the desired mm-wave bands. In addition, the low profile of the proposed IRS has made it applicable to conformal surfaces. The IRS prototype consisting of 32 $\times$ 32 unit-cells with a total size of 118 $\times$ 118 mm$^2$ has been fabricated and measured. The transmission coefficient S21 $<-$ 10 dB is achieved from 20 to 26 GHz and 37 to 41.8 GHz with a fractional bandwidth of B.W. of 27 % and 10.5 %, respectively, and reflection coefficient S11 $<-$ 10 dB is achieved from 29.5 to 31.2 GHz. The measured results show good agreement with the simulated results. The overall performance of the proposed passive IRS makes it an attractive candidate for coverage enhancement in 5G and beyond communications.

## Data Availability

The datasets used and/or analysed during the current study available from the corresponding author on reasonable request.
